# Risk of ischemic stroke after atrial fibrillation diagnosis: A national sample cohort

**DOI:** 10.1371/journal.pone.0179687

**Published:** 2017-06-21

**Authors:** Mi Kyoung Son, Nam-Kyoo Lim, Hyung Woo Kim, Hyun-Young Park

**Affiliations:** Division of Cardiovascular and Rare Diseases, Center for Biomedical Science, Korea National Institute of Health, Chungcheongbuk-do, Republic of Korea; University of Miami School of Medicine, UNITED STATES

## Abstract

Atrial fibrillation (AF) is a major risk factor for ischemic stroke and associated with a 5-fold higher risk of stroke. In this retrospective cohort study, the incidence of and risk factors for ischemic stroke in patients with AF were identified. All patients (≥30 years old) without previous stroke who were diagnosed with AF in 2007–2013 were selected from the National Health Insurance Service-National Sample Cohort. To identify factors that influenced ischemic stroke risk, Cox proportional hazard regression analysis was conducted. During a mean follow-up duration of 3.2 years, 1022 (9.6%) patients were diagnosed with ischemic stroke. The overall incidence rate of ischemic stroke was 30.8/1000 person-years. Of all the ischemic stroke that occurred during the follow-up period, 61.0% occurred within 1-year after AF diagnosis. Of the patients with CHA_2_DS_2_-VASc score of ≥2, only 13.6% were receiving warfarin therapy within 30 days after AF diagnosis. Relative to no antithrombotic therapy, warfarin treatment for >90 days before the index event (ischemic stroke in stroke patients and death/study end in non-stroke patients) associated with decreased ischemic stroke risk (Hazard Ratio = 0.41, 95%confidence intervals = 0.32–0.53). Heart failure, hypertension, and diabetes mellitus associated with greater ischemic stroke risk. AF patients in Korea had a higher ischemic stroke incidence rate than patients in other countries and ischemic stroke commonly occurred at early phase after AF diagnosis. Long-term (>90 days) continuous warfarin treatment may be beneficial for AF patients. However, warfarin treatment rates were very low. To prevent stroke, programs that actively detect AF and provide anticoagulation therapy are needed.

## Introduction

Atrial fibrillation (AF) is one of the most common sustained arrhythmias [1]. It is an important risk factor for ischemic stroke since it associates with a 5-fold higher risk of stroke compared with the general population [2]. In patients with non-valvular AF, anticoagulation therapy (OAC) with warfarin reduces stroke by 64% compared with no antithrombotic agents. OAC is significantly more effective in reducing stroke risk in AF than antiplatelet agents [3]. American College of Cardiology/American Heart Association/Heart Rhythm Society (ACC/AHA/HRS) guidelines and the Korean Heart Rhythm Society (KHRS) recommend OAC therapy for patients with AF who are at moderate or high risk of stroke (*i*.*e*., have a CHA_2_DS_2_-VASc score of ≥1) [4,5]. However, OAC therapy is underused in AF: only 50% (USA), 67% (Europe), 75% (Japan), and 2.5% (China) of AF patients with CHADS_2_ or CHA_2_DS_2_-VASc scores of ≥2 receive OAC therapy. This reflects its narrow therapeutic range and/or its association with complications [6].

Stroke is a major health burden in Korea: indeed, it is the second leading cause of death after cancer. Moreover, of all Organization for Economic Cooperation and Development countries, Korea is experiencing the fastest aging of the population. Thus, the burden of AF and stroke is expected to rise further.

Race/ethnicity influences the risk of stroke in patients with AF [7,8]: in whites, blacks, Hispanics, and Asians who are treated with warfarin, the ischemic stroke rates per 1000 person-years’ follow-up are 15.9, 27.1, 15.1, and 13.4, respectively [8], while the equivalent rates for subjects not using warfarin are 20.3, 29.4, 21.3, and 20.6 strokes per 1000 person-years, respectively. Moreover, Healey *et al*. showed that rates of stroke occurrence within a year of presenting with AF at a hospital emergency department according to the countries were as follows: North America, Western Europe, or Australia (2%), South America (3%), Eastern Europe (4%), Southeast Asia (7%), China (7%), Africa (8%), and India (0.8%). The incidence of all-causes death within 1 year after presenting with AF also varies widely between these regions from 9% to 20% [7].

In Korea, there is limited information about the risk of stroke in patients with AF: only a few epidemiological studies have assessed the prevalence or incidence of AF in Korea [9–11]. To develop a strategic plan for stroke prevention, it is necessary to know the incidence and risk of stroke in Korean patients with AF. Therefore, this large population-based cohort study investigated the incidence and risk of ischemic stroke in patients with AF with various ages and CHA_2_DS_2_-VASc scores. The effect of OAC therapy on ischemic stroke incidence and risk was also assessed.

## Materials and methods

### Data source

This study was conducted using the database of the Korean National Health Insurance Service-National Sample Cohort (NHIS-NSC). This database consists of approximately one million medical insurance subscribers who were selected from the NHIS database using a stratified random sampling method that involved 1476 strata, namely, sex (two strata), age (18 strata), and level of income (41 strata) [12]. In 2002, the subjects in the NHIS-NSC database constituted 2.2% of the entire South Korean population. The subscribers were followed for 11 years (*i*.*e*., until 2013) unless the subscriber was no longer considered eligible for health insurance due to death or emigration. During the follow-up period, the cohort was refreshed annually by adding a representative sample of newborns that were sampled using 82 strata (two for sex and 41 for parental income levels) with a sampling rate of 2.2%. The NHIS-NSC database includes demographic information, medical claims data, and disease diagnoses that were defined according to the Korean Classification of Diseases-6 (KCD-6), which is similar to the International Classification of Diseases-10 (ICD-10). The study protocol was approved by the institutional review board of the Health Insurance Review and Assessment Service. Written informed consent was not required as the database maintained de-identification and anonymity of sampled individuals.

### Study population and study outcomes

The NHIS-NSC database was available for the years 2002–2013. The study cohort consisted of all patients aged ≥30 years who had their first inpatient or outpatient diagnosis of AF between January 1, 2007 and December 31, 2013. AF was defined as a diagnosis of AF or atrial flutter (KCD-6: I48).

The NHIS-NSC database was used to determine whether the study patients had an ischemic stroke after the AF diagnosis. Ischemic stroke was defined as ischemic cerebrovascular disease, including transient ischemic attack and retinal infarction [KCD-6: I63, I64, G45 (excluding G45.4), and H34.1], as previously reported [13]. To minimize the inclusion of patients with recurrent strokes, all patients who had an ischemic stroke less than 6 years before AF was diagnosed were excluded. In other words, the included patients were only those who had not had an ischemic stroke within the 6 years before they received the AF diagnosis. Thus, the ischemic strokes that occurred in the study cohort during follow-up after AF diagnosis were considered to be first-time ischemic strokes. In addition, to overcome the impact of rule-out diagnoses and to improve the specificity of our definition of ischemic stroke, only the patients who were hospitalized for more than 2 days for ischemic stroke, or who continuously visited the hospital for more than 2 days for ischemic stroke, were considered to definitely have had an ischemic stroke.

The patients in the study cohort were followed until index date (ischemic stroke, death, or the end of the study period [December 31, 2013], whichever came first).

Whether the study subjects had comorbidities of AF was determined by searching retrospectively for the KCD-6 disease codes. The comorbidities that were assessed were heart failure, hypertension, diabetes mellitus, ischemic heart disease (IHD), valvular heart disease (VHD), cardiomyopathy, and chronic kidney disease (CKD).

The patients were divided into five categories on the basis of their CHA_2_DS_2_-VASc score at AF diagnosis (scores of 0, 1, 2, 3–4, and 5–7). The patients were also divided into six age groups (30–39, 40–49, 50–59, 60–69, 70–79, and ≥80 years).

### Warfarin exposure

Exposure to warfarin was assessed using an algorithm that was suggested by Azoulay *et al*. and Go *et al*. [14,15]. Patients were assessed for warfarin exposure period as the prescription coverage plus 45-days (30-days grace period plus 15-days elimination period). Patients were considered to be continuously exposed to warfarin if each warfarin exposure overlapped, that is, continuous warfarin exposure was assumed for periods where the second of any 2 consecutive filled prescriptions began within 45 days of the last day supplied by the previous prescription. The duration of continuous warfarin therapy was thus the duration between the start date and the end date of the total period of continuous warfarin exposure (S1 Fig).

Warfarin treatment was defined as “current” treatment if the warfarin exposure period overlapped the index date. Similarly, antiplatelet treatment was considered to be current if it overlapped the index date. The patients were classified into the following four mutually exclusive groups on the basis of their warfarin and antiplatelet exposure at the index date (S2 Fig): (1) current use of continuous warfarin therapy was initiated ≤90 days before the index date, (2) current use of continuous warfarin therapy was initiated >90 days before the index date, (3) current use of antiplatelet therapy only, and (4) no current use of any antithrombotic therapy.

Through these algorithms, the proportions of patients who received warfarin therapy within 30 days after AF diagnosis and whose warfarin therapy continued during follow-up were measured, and the risk of ischemic stroke in patients with AF by current use of warfarin therapy was also measured.

### Statistical analysis

All variables were categorical and were expressed as frequency and percentage. The patients who did and did not have a first-time ischemic stroke were compared in terms of their demographic, CHA_2_DS_2_-VASc scores, comorbidities of AF and their current use of antithrombotic therapy at the index date using Chi-squared tests.

The incidence of ischemic stroke in the whole cohort, in males and females, in specific age subgroups, and in specific CHA_2_DS_2_-VASc score subgroups was calculated. Incidence of ischemic stroke after AF diagnosis was computed as the number of patients with stroke during the follow-up period divided by the total person-years at risk and was reported as cases per 1000 person-years. Kaplan-Meier survival curves were used to estimate the rate of continuous warfarin treatment.

To identify risk factors for ischemic stroke in patients with AF, Cox’s proportional hazard regression analyses were performed after adjusting for potential confounders. Hazard ratios (HRs) and 95% confidence intervals (CIs) were estimated.

All statistical tests were two-tailed, and *p*-values of <0.05 were considered statistically significant. All statistical analyses were performed using SAS software (ver. 9.4; SAS Institute, Cary, NC, USA).

## Results

In total, 14594 subjects in the database were diagnosed with AF for the first time in 2007–2013. Of these, 3940 were excluded because they had an ischemic stroke less than 6 years before their AF diagnosis. Thus, the study cohort consisted of 10654 subjects who were diagnosed with first-time AF during the study period. The majority of patients (68.9%) were between 50 and 79 years of age. Half (53.6%) were male. The most common CHA_2_DS_2_-VASc score was 3–4 (36.3%), and 78.3% of the patients had scores between 1 and 4. In terms of the specific risk components in the CHA_2_DS_2_-VASc score, the majority of patients had hypertension (69.9%), a third had diabetes mellitus (36.9%), and a quarter were ≥75 years of age (26.9%). Among patients with AF before index date, 39% had IHD, 9% had VHD, 6.5% had CKD, and 3.1% had cardiomyopathy. At the index date, the majority (58.8%) of patients were not treated with any antithrombotic therapy. The remaining patients were treated most commonly with current antiplatelet therapy (29.4%), followed by current continuous warfarin therapy that started >90 days before the index date (10.6%) and current continuous warfarin therapy that started ≤90 days before the index date (1.2%) (Table 1).

**Table 1:** Characteristics of the study population who did and did not develop stroke during follow-up.

Characteristic	Overall (*n* = 10654)	Incident stroke		*P*-value
		Yes (*n* = 1022)	No (*n* = 9632)	
**Age at AF diagnosis**, years				<0.001
30–39	691 (6.5)	8 (0.8)	683 (7.1)	
40–49	1284 (12.1)	42 (4.1)	1242 (12.9)	
50–59	2150 (20.2)	131 (12.8)	2019 (21.0)	
60–69	2575 (24.2)	249 (24.4)	2326 (24.1)	
70–79	2613 (24.5)	380 (37.2)	2233 (23.2)	
≥80	1341 (12.6)	212 (20.7)	1129 (11.7)	
**Sex**				0.113
Male	5713 (53.6)	524 (51.3)	5189 (53.9)	
Female	4941 (46.4)	498 (48.7)	4443 (46.1)	
**CHA**_**2**_**DS**_**2**_**-VASc score at AF diagnosis**				<0.001
0	857 (8.0)	21 (2.1)	836 (8.7)	
1	2150 (20.2)	85 (8.3)	2065 (21.4)	
2	2326 (21.8)	177 (17.3)	2149 (22.3)	
3–4	3836 (36.3)	509 (49.8)	3359 (34.9)	
5–7	1453 (13.6)	230 (22.5)	1223 (12.7)	
**Specific components of CHA**_**2**_**DS**_**2**_**-VASc score**				
Congestive heart failure/left ventricular dysfunction	2716 (25.5)	336 (32.9)	2380(24.7)	<0.001
Hypertension	7442 (69.9)	879 (86.0)	6563 (68.1)	<0.001
Age ≥75 years	2871 (26.9)	369 (36.1)	2502 (26.0)	<0.001
Diabetes mellitus	3929 (36.9)	472 (46.2)	3457 (35.9)	<0.001
History of vascular disease	1081 (10.1)	110 (10.8)	971 (10.1)	0.492
Age 65–74 years	2480 (23.3)	386 (37.8)	2094 (21.7)	<0.001
**Comorbidity of AF**				
IHD	4160 (39.0)	450 (44.0)	3710 (38.5)	0.001
VHD	954 (9.0)	103 (10.1)	851 (8.8)	0.186
Cardiomyopathy	325 (3.1)	33 (3.2)	292 (3.0)	0.727
CKD	694 (6.5)	71 (6.9)	623 (6.5)	0.555
**Current use of antithrombotic therapy**				<0.001
No antithrombotics	6269 (58.8)	645 (63.1)	5624 (58.4)	
Continuous warfarin therapy ≤90 days	130 (1.2)	18 (1.8)	112 (1.2)	
Continuous warfarin therapy >90 days	1128 (10.6)	76 (7.4)	1052 (10.9)	
Antiplatelet therapy only	3127 (29.4)	283 (27.7)	2844 (29.5)	

The data are reported as *n* (%).

Abbreviations: CKD, chronic kidney disease; IHD, ischemic heart disease; TIA, transient cerebral ischemic attack; VHD, valvular heart disease.

*P-*values were determined using Chi-squared tests or Fisher’s exact test.

Current use indicates the treatment (or lack thereof) at the time of the index event. The index event date was the date of ischemic attack in the stroke group and the date of death or the end of the study period (December 31, 2013) in the non-stroke group.

Warfarin treatment was defined as continuous when the warfarin exposure periods (prescription coverage plus 45 days) overlapped each other.

### Incidence of ischemic stroke after AF diagnosis

The mean follow-up duration of the cohort was 3.2 (range, 1 days to 7 years) years. During follow-up, 1022 (9.6%) of the patients were diagnosed with first-time ischemic stroke. Of these, 524 (51.3%) were males and 498 (48.7%) were females. Similar gender frequencies were observed in the non-stroke patients. Compared with the non-stroke patients, the stroke patients were older and had higher CHA_2_DS_2_-VASc scores at the time of AF diagnosis (89.6% had CHA_2_DS_2_-VASc scores of ≥2, compared with 69.9% in the non-stroke group). In terms of specific CHA_2_DS_2_-VASc score components, the stroke patients were significantly more likely to have hypertension (86.0% *vs*. 68.1%), diabetes mellitus (46.2% *vs*. 35.9%), and congestive heart failure (32.9% *vs*. 24.7%). In terms of comorbidities, the stroke patients were more likely to have IHD (44.0 *vs*. 38.5%). The rates of other comorbidities were similar between the two groups. The stroke patients were more likely to not be taking any antithrombotic therapies at the index event (63.1% *vs*. 58.4%) and less likely to be taking antiplatelet therapy alone (27.7% *vs*. 29.5%) or warfarin therapy (9.2% *vs*. 12.1%) (Table 1).

The overall incidence of ischemic stroke in the study cohort during the follow-up period was 30.8/1000 person-years (Table 2). Subgroup analyses showed that the incidence rose substantially with increasing age (it was 3.1/1000 person-years in the 30–39 year age group *vs*. 76.0/1000 person-years in the ≥80 year age group) and increasing CHA_2_DS_2_-VASc score (scores of 0, 1, 2, 3–4, and 5–7 associated with incidences of 7.0, 11.1, 22.4, 45.6, and 66.9/1000 person-years, respectively). The incidence of ischemic stroke in patients who were not taking antithrombotic therapy at the index event was 38.4/1000 person-years. In the patients taking antiplatelet therapy only, this incidence was 28.5/1000 person-years. In the patients taking continuous warfarin therapy for >90 days and ≤90 days before the index event, the incidences were 16.7 and 122.0/1000 person-years, respectively. Thus, the lowest incidence of ischemic stroke was in the group who received continuous warfarin therapy starting >90 days before the index event.

**Table 2:** Incidence per 1000 person-years of ischemic stroke after diagnosis of atrial fibrillation.

Characteristic	Cases (%)	Person-year	IR (95% CI)
**Overall**	1022 (9.6)	33164.24	30.82 (28.96–32.73)
**Age at AF diagnosis**, years			
30–39	8 (1.2)	2616.02	3.06 (1.32–5.51)
40–49	42 (3.3)	4701.65	8.93 (6.44–11.83)
50–59	131 (6.1)	7319.04	17.90 (14.96–21.09)
60–69	249 (9.7)	8718.04	28.56 (25.12–32.22)
70–79	380 (14.5)	7020.87	54.12 (48.68–59.55)
≥80	212 (15.8)	2788.62	76.02 (66.13–86.59)
**Sex**			
Male	524 (9.2)	17689.76	29.62 (27.14–32.21)
Female	498 (10.1)	15474.48	32.18 (29.42–35.07)
**CHA**_**2**_**DS**_**2**_**-VASc score at AF diagnosis**			
0	21 (2.5)	2999.07	7.00 (4.33–10.30)
1	85 (4.0)	7656.59	11.10 (8.87–13.58)
2	177 (7.6)	7906.90	22.39 (19.21–25.80)
3–4	509 (13.2)	11163.58	45.59 (41.72–49.64)
5–7	230 (15.8)	3438.10	66.90 (58.53–75.82)
**Current use of antithrombotic therapy**			
No antithrombotics	645 (10.3)	18531.55	34.81 (32.17–37.54)
Continuous warfarin ≤90 days	18 (13.8)	147.55	121.99 (72.30–184.47)
Continuous warfarin >90 days	76 (6.7)	4553.05	16.69 (13.15–20.65)
Antiplatelet therapy only	283 (9.1)	9932.10	28.49 (25.27–31.91)

Abbreviations: CI, confidence interval; IR, incidence rate.

Current use indicates the treatment (or lack thereof) at the time of the index event. The index event date was the date of ischemic attack in the stroke group and the date of death or the end of the study period (December 31, 2013) in the non-stroke group.

Warfarin treatment was defined as continuous when warfarin exposure periods (prescription coverage plus 45 days) overlapped each other.

**Fig 1:**
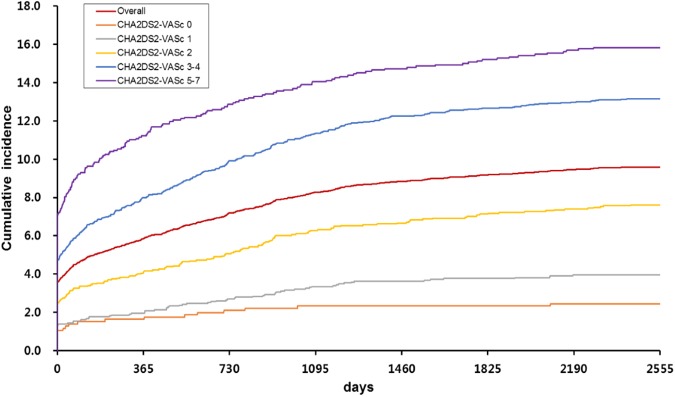
Cumulative incidence of ischemic stroke over time after atrial fibrillation diagnosis in the whole cohort and the CHA_2_DS_2_-VASc score subgroups.

Fig 1 shows the cumulative incidence of ischemic stroke during follow-up. Of all the ischemic strokes that occurred during the follow-up period, 61.0% occurred during the first year after AF diagnosis. Similarly, of the ischemic strokes that occurred in the patients with CHA_2_DS_2_-VASc scores at AF diagnosis of 0, 1, 2, 3–4, and 5–7, the majority occurred in the first year after AF diagnosis (66.7%, 49.4%, 53.7%, 60.7%, and 70.9%, respectively). After the first year, the cumulative incidences tended to plateau. As expected from Table 2, greater CHA_2_DS_2_-VASc scores associated with greater cumulative incidences of ischemic stroke (Fig 1).

### Warfarin therapy rates during follow-up after AF diagnosis

#### Low rates of warfarin therapy after AF diagnosis

**Fig 2:**
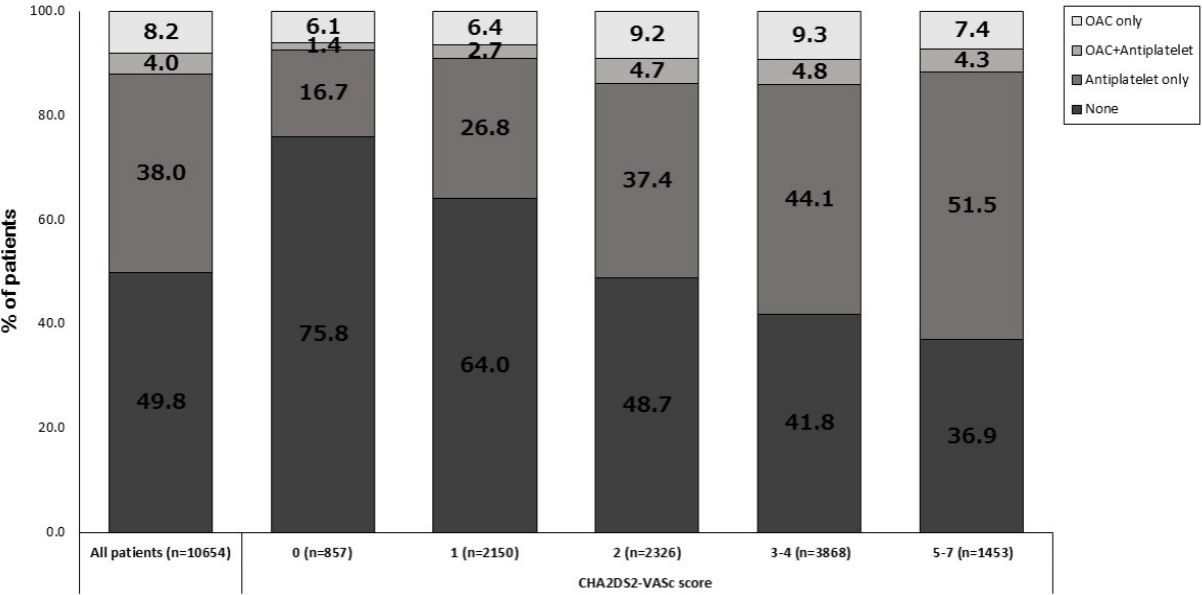
Proportion of anticoagulation therapy (warfarin) in patients within 30 days after atrial fibrillation diagnosis in the CHA_2_DS_2_-VASc score subgroups.

**Fig 3:**
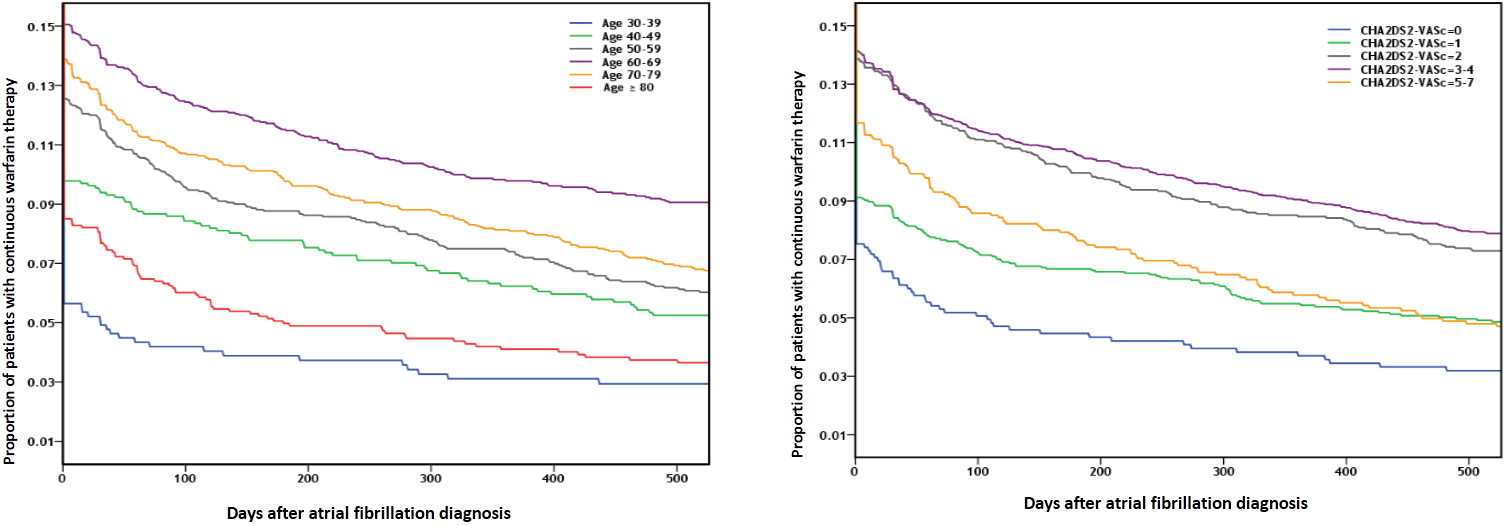
Proportion of continuous warfarin therapy after atrial fibrillation diagnosis in (A) specific age subgroups and (B) CHA_2_DS_2_-VASc score subgroups.

Only 12.2% of all patients were being prescribed warfarin therapy within 30 days after AF diagnosis (Fig 2). Even in patients with CHA_2_DS_2_-VASc scores of ≥2, the warfarin treatment rate was only 13.6%. This situation worsened over time after AF diagnosis: only 10.1% and 7.2% of all patients diagnosed with AF were receiving continuous warfarin therapy 3 months and 1 year after AF diagnosis, respectively. The 60–69-year-olds had the highest rate of warfarin treatment (15.0%) while the youngest patients (30–39 years of age) had the lowest rate (5.6%). The other age groups had intermediate rates. Similarly, 3 months after AF diagnosis, 13.0% and only 4.2% of the 60–69- and 30–39-year-olds were receiving continuous warfarin treatment, respectively (Fig 3A). Notably, approximately 20% and 80% of the patients aged 60–69 and 30–39 years had a CHA_2_DS_2_-VASc score of ≤1 at AF diagnosis, respectively (S1 Table). These observations suggest that low CHA_2_DS_2_-VASc scores were partly responsible for the low rates of warfarin treatment in the youngest group after AF diagnosis.

Within 30 days after AF diagnosis, 7.5%, 9.1%, 13.9%, 13.1%, and 11.7% of the patients with CHA_2_DS_2_-VASc scores of 0, 1, 2, 3–4, and 5–7 were treated with warfarin, respectively. Moreover, 3 months after AF diagnosis, 5.2%, 7.4%, 11.3%, 11.6%, and 8.8% had continuous warfarin therapy, respectively (Fig 3B). Thus, the patients with CHA_2_DS_2_-VASc scores of 2 and 3–4 had the highest rates of warfarin treatment at various time points after AF diagnosis.

#### Warfarin treatment continuation after AF diagnosis

Of the 1299 patients who were receiving warfarin therapy within 30 days after AF diagnosis, 24.6% of the patients with a CHA_2_DS_2_-VASc score of 5–7 had discontinued warfarin therapy within 3 months. The patients with a CHA_2_DS_2_-VASc score of 0 showed even higher discontinuation rates within 3 months (31.3%). By contrast, the other three subgroups with CHA_2_DS_2_-VASc scores of 1, 2, and 3–4 exhibited lower rates of discontinuation within 3 months (19.4%, 18.7%, and 18.3%, respectively) (S3(A) Fig).

The youngest (30–39 years) and oldest (≥80 years) patients were more likely than the other age groups to discontinue warfarin, with 25.6% and 27.5%, respectively, having discontinued warfarin within the third month after AF diagnosis (S3(B) Fig).

#### Risk factors for ischemic stroke

Table 3 shows the risk factors for incident ischemic stroke after adjusting for variables that relate to incident ischemic stroke. An age of ≥75 years and the presence of specific components of the CHA_2_DS_2_-VASc score (congestive heart failure, hypertension, and diabetes mellitus) associated significantly with increased risk of ischemic stroke in both univariable and multivariable analyses (p<0.05 each). The strongest risk factor for incident ischemic stroke was hypertension (HR = 2.71, 95% CI = 2.25–3.26). Relative to non-use of antithrombotic therapy, continuous warfarin therapy starting >90 days (HR = 0.41, 95% CI = 0.32–0.53) and antiplatelet therapy (HR = 0.64, 95% CI = 0.56–0.74) before index date decreased the risk of ischemic stroke. By contrast, continuous warfarin therapy starting ≤90 days before index date did not associate significantly with ischemic stroke risk (HR = 1.48, 95% CI = 0.93–2.38).

**Table 3:** Factors that influenced the risk of incident stroke in patients with atrial fibrillation.

Variables	Univariable		Multivariable	
	HR (95% CI)	*P*-value	HR (95% CI)	*P*-value
**Female**	1.091 (0.965–1.234)	0.163	1.036 (0.915–1.172)	0.577
**Age**, ≥75 years	1.551 (1.365–1.762)	<0.001	1.447 (1.271–1.647)	<0.001
**Comorbidity of AF**				
Congestive heart failure	1.471 (1.291–1.676)	<0.001	1.231 (1.072–1.413)	0.003
Hypertension	2.817 (2.360–3.361)	<0.001	2.705 (2.245–3.259)	<0.001
Diabetes mellitus	1.629 (1.440–1.842)	<0.001	1.363 (1.200–1.549)	<0.001
IHD	1.263 (1.116–1.429)	0.001	1.014 (0.890–1.154)	0.838
VHD	1.071 (0.874–1.313)	0.507	1.129 (0.912–1.399)	0.266
Cardiomyopathy	1.063 (0.752–1.504)	0.728	0.886 (0.624–1.257)	0.497
CKD	1.150 (0.904–1.464)	0.255	0.867 (0.679–1.109)	0.256
**Current use of antithrombotic therapy**				
No antithrombotics	Reference		Reference	
Continuous warfarin ≤90 days	2.023 (1.265–3.235)	0.003	1.482 (0.925–2.375)	0.102
Continuous warfarin >90 days	0.549 (0.432–0.696)	<0.001	0.411 (0.321–0.526)	<0.001
Antiplatelet therapy only	0.837 (0.728–0.962)	0.013	0.642 (0.556–0.741)	<0.001

Abbreviations: CI, confidence interval; CKD, chronic kidney disease; HR, hazard ratio; IHD, ischemic heart disease; VHD, valvular heart disease.

The *P*-values were obtained by Cox proportional hazard regression analysis. Current use indicates the treatment (or lack thereof) at the time of the index event. The index event date was the date of ischemic attack in the stroke group and the date of death or the end of the study period (December 31, 2013) in the non-stroke group.

Warfarin treatment was defined as continuous when warfarin exposure periods (prescription coverage plus 45 days) overlapped each other.

## Discussion

The results of this large population-based study indicate that, in patients with AF, the incidence of ischemic stroke increased with age and CHA_2_DS_2_-VASc score and the risk factors for ischemic stroke were age, heart failure, hypertension, and diabetes mellitus. Moreover, the majority of ischemic strokes during follow-up occurred within the first year after AF diagnosis. In addition, long-term (at least 90 days) warfarin therapy associated strongly with a decreased risk of ischemic stroke. However, the warfarin treatment rate in the study population was very low.

Stroke is the second leading cause of death after cancer and the first leading cause of death that is due to a single organ disease in South Korea. AF is one of the most important risk factors for stroke because it associates with a 4- to 5-fold higher risk of stroke relative to the general population [2]. Notably, the prevalence of AF in South Korea increased between 2008 and 2015 from 0.40% to 0.67% [16]. It is likely that AF prevalence will increase due to the aging of the South Korean population and the increased frequency of AF comorbidities such as hypertension and diabetes mellitus. These observations suggest that ischemic stroke in patients with AF will become a major public health problem in South Korea in the future.

The overall incidence of ischemic stroke in our patients with AF (30.8 per 1000 person-years) was higher than the ischemic stroke incidences in AF that were reported in other countries [7,17–19]: the ACTIVE W (Atrial Fibrillation Clopidogrel Trial With Irbesartan for Prevention of Vascular) study in Canada reported that the annualized risk of stroke in paroxysmal and sustained AF was 20 and 22 per 1000 person-years, respectively [17]. In Denmark, 11.28% of AF patients had a diagnosis of incident stroke in 1980–2002 [20]. In Taiwan and Japan, the ischemic stroke rate in AF patients was 28.4 and 13.3 per 1000 person-years, respectively [18,19], while the analysis of Swedish Hospital Discharge and Cause of Death Registries showed that, within 3 years of hospitalization with AF, 7.7% were diagnosed with ischemic stroke [21]. Our higher incidence of ischemic stroke may reflect the different race/ethnicity of our patients compared with those in the other studies. That race/ethnicity differences influence ischemic stroke rates in AF has been observed previously [7,8]. For example, Healey *et al*. showed that ischemic stroke rates of patients with AF varied between countries: the highest and lowest frequencies of strokes were found in patients in Africa (8%) and in India (<1%), respectively [7]. Another contributing factor may be our low OAC (warfarin) treatment rate. In the USA, Europe, Japan, and China, 50%, 67%, 75%, and 2.5% of AF patients with CHADS_2_ or CHA_2_DS_2_-VASc scores of ≥2 receive OAC therapy, respectively [6]. Our treatment rate of patients with scores of ≥2 was only 13.6%.

In the present study, the incidence of ischemic stroke in the first year after AF diagnosis was 6%. This rose to 9.6% at the end of the follow-up period. Thus, 60% of all strokes occurred in the first year after AF diagnosis. This indicates that it is essential to institute treatment for AF early. Similarly, the cohort study of Healey *et al*., which employed a prospective registry of patients in 47 countries, reported that, 1 year after AF diagnosis, about 4% of patients have had a stroke and about 11% of these patients die [7]. ACC/AHA/HRS and KHRS guidelines recommend that AF patients with a CHA_2_DS_2_-VASc score ≥1 should receive ongoing thromboembolic prophylaxis with OAC therapy [4,5]. While OAC therapy with warfarin, which is a synthetic derivative of coumarin, associates with more frequent and costly monitoring and a higher risk of hemorrhage than other treatments, it very effectively reduces stroke risk (by 64–70%) [3]. Notably, the UK Clinical Practice Research Datalink reported that, while warfarin treatment for more than 30 days associates with a decreased risk of ischemic stroke in AF patients, warfarin treatment for less than 30 days associates with an increased risk of ischemic stroke due to the transient hypercoagulable state at the start of warfarin treatment [22]. Another study also showed that the net clinical benefit of warfarin only becomes apparent after 3 months of continuous use [14]. Similarly, the present study showed that the use of warfarin therapy for more than 3 months before the index date effectively reduced the risk of ischemic stroke, whereas the use of warfarin therapy for less than 3 months did not associate with a change in risk of ischemic stroke.

The warfarin treatment rate in our Korean patients with AF was very low (12.2% overall). Particularly low warfarin treatment rates were observed in the oldest and youngest patients (≥80 and 30–39 years, respectively) who had CHA_2_DS_2_-VASc scores of ≥2 (S4 Fig). The reasons for warfarin underuse included monitoring difficulties, concerns about the risk of bleeding complications, and past experiences with antithrombotic agents [23,24]. Indeed, Hsu *et al*. reported that AF with coronary atherosclerosis-related comorbidities associated with more frequent prescription of aspirin instead of OAC prescription [25]. It has been observed that antiplatelet agents also effectively reduce stroke risk (by 30–50%) [26]. In the present study, 29.4% of the patients were on antiplatelet therapy at the time of the index event. The rates of antiplatelet therapy in the patients who did and did not have ischemic stroke during follow-up were 27.7% and 29.5%, respectively, and antiplatelet therapy at the time of the index event significantly reduced the risk of ischemic stroke. However, warfarin therapy clearly reduced the risk of ischemic stroke more effectively than antiplatelet agents (HR = 0.41, 95% CI = 0.32–0.53 *vs*. HR = 0.64, 95% CI = 0.56–0.74). This is consistent with previous reports [3,26]. Recently, novel oral anticoagulant drugs (NOACs) were found to be as effective, safe, and convenient as OACs [27–30]. Since NOACs have been reimbursed by the Korean National Health Insurance since 2015, it is likely that their use in South Korea will increase, including in AF patients with relatively low CHA_2_DS_2_-VASc scores.

The multivariable analyses in this study showed that an older age (≥75 years) and the presence of heart failure, hypertension, and diabetes mellitus associated significantly with increased risk of ischemic stroke. These observations were reported previously [19,21,31]. We found that the strongest risk factor for incident ischemic stroke was hypertension. However, we failed to find an association between sex and the incidence of ischemic stroke in AF. This is inconsistent with previous reports [32,33]. Moreover, we did not find that IHD and CKD increased the risk of incident ischemic stroke. By contrast, the Swedish and Danish national registries reported that IHD and CKD increase the risk of stroke in AF, respectively [21,34]. We also failed to detect an association between VHD and cardiomyopathy and increased risk of stroke in AF. By contrast, Sacco *et al*. found that both conditions associated with an increased stroke risk [31].

This study had several limitations. First, ischemic stroke, CHA_2_DS_2_-VASc score, and comorbid conditions were defined on the basis of KCD-6 codes. Since claims data are prone to errors such as miscoding, this may have introduced classification bias into the study. To overcome these limitations, we used a strict definition of ischemic stroke, namely, that the patients who had only received one inpatient/outpatient diagnosis of ischemic stroke were not considered to have definitely had an ischemic stroke. This minimized the impact of rule-out diagnoses and improved the specificity of our definition of ischemic stroke. Second, the nature of the drug information in the NHIS-NSC database means that it is not known whether the patients actually used warfarin according to the prescription. Thus, it is possible that some unexposed patients were misclassified as exposed. This would have the effect of underestimating the treatment effects. Third, we were unable to determine the international normalized ratio (INR) levels of the patients. INR is a laboratory value that is used to monitor the therapeutic range of warfarin. Instead, we considered the warfarin exposure period including the grace and elimination periods (namely, the prescription coverage plus 45-days).

Despite these limitations, this study had several strengths. First, we used the NHIS-NSC database, which contains a large sample cohort that is based on nationwide health insurance data. It is representative of the population and overcomes the limitations of cross-sectional data. Second, to our knowledge, this study is the first to investigate the incidence of ischemic stroke in patients with AF in Korea and the association between the current use of warfarin and the incidence of ischemic stroke in these patients.

## Conclusions

This nationwide survey on the incidence of ischemic stroke in patients with AF was based on medical claim data in the NHIS-NSC database. It showed that the incidence of ischemic stroke increased as the age and CHA_2_DS_2_-VASc score rose. The overall incidence was higher than that reported in other countries. The majority of ischemic strokes occurred in the first year after AF. Thus, early treatment after AF diagnosis is essential. Long-term (>90 days) warfarin therapy before the index event effectively reduced the risk of ischemic stroke. However, the warfarin treatment rate in the cohort was very low. This suggests that warfarin therapy of AF patients in South Korea should be increased. Although antiplatelet therapy was less effective than warfarin, it also effectively reduced the risk of ischemic stroke, although additional studies are needed to confirm these findings. To prevent stroke after AF diagnosis, programs that actively detect AF early and effectively manage AF are needed.

## Supporting information

S1 Fig The estimation of continuous warfarin exposure. (PDF)

S2 Fig Current use of continuous antithrombotic therapy. (PDF)

S3 Fig Proportion of the patients receiving warfarin after atrial fibrillation diagnosis whose warfarin therapy continued during follow-up. (A) CHA_2_DS_2_-VASc score and (B) age subgroups. Warfarin treatment was defined as continuous when the warfarin exposure periods (prescription coverage plus 45 days) overlapped each other.
(PDF)

S4 Fig Proportion of patients with CHA_2_DS_2_-VASc scores of ≥2 who received continuous warfarin therapy after atrial fibrillation diagnosis. (PDF)

S1 Table Frequency of patients in each age subgroup with CHA_2_DS_2_-VASc scores of 0, 1, 2, 3–4, and 5–7 at atrial fibrillation diagnosis. The data are reported as *n* (%).
(PDF)
