# Supplementary material for: Risk of ischemic stroke after atrial fibrillation diagnosis: A national sample cohort
Source: PLoS One. 2017 Jun 21;12(6):e0179687. doi: 10.1371/journal.pone.0179687 (PMC5479557; doi:10.1371/journal.pone.0179687)
Supplement: S1 Fig — (PDF) [file pone.0179687.s001.pdf]

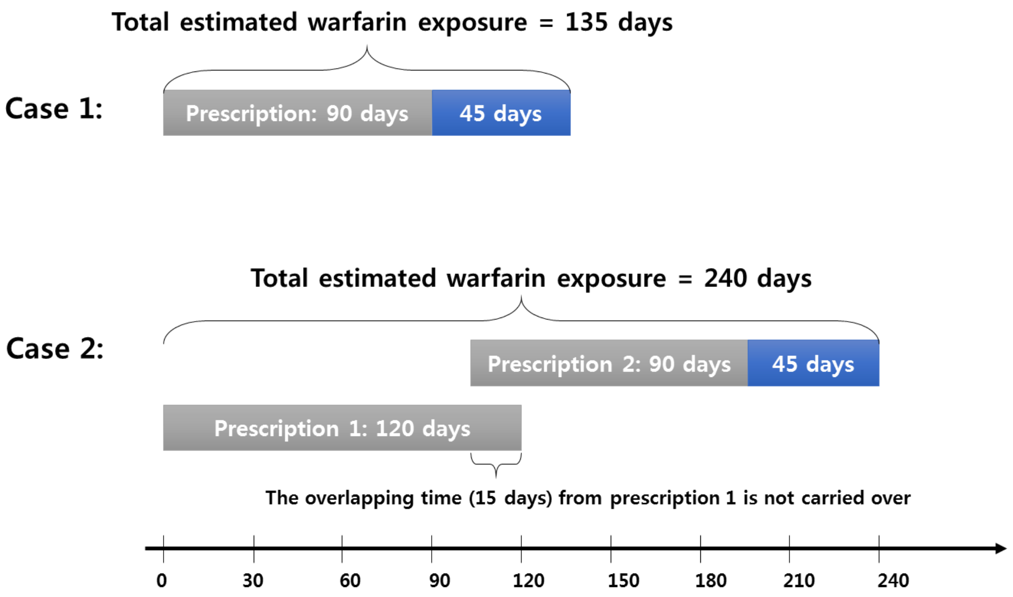

S1 Fig. The estimation of continuous warfarin exposure
