# Supplementary figures and images for: Risk of ischemic stroke after atrial fibrillation diagnosis: A national sample cohort
Source: PLoS One. 2017 Jun 21;12(6):e0179687. doi: 10.1371/journal.pone.0179687 (PMC5479557; doi:10.1371/journal.pone.0179687)

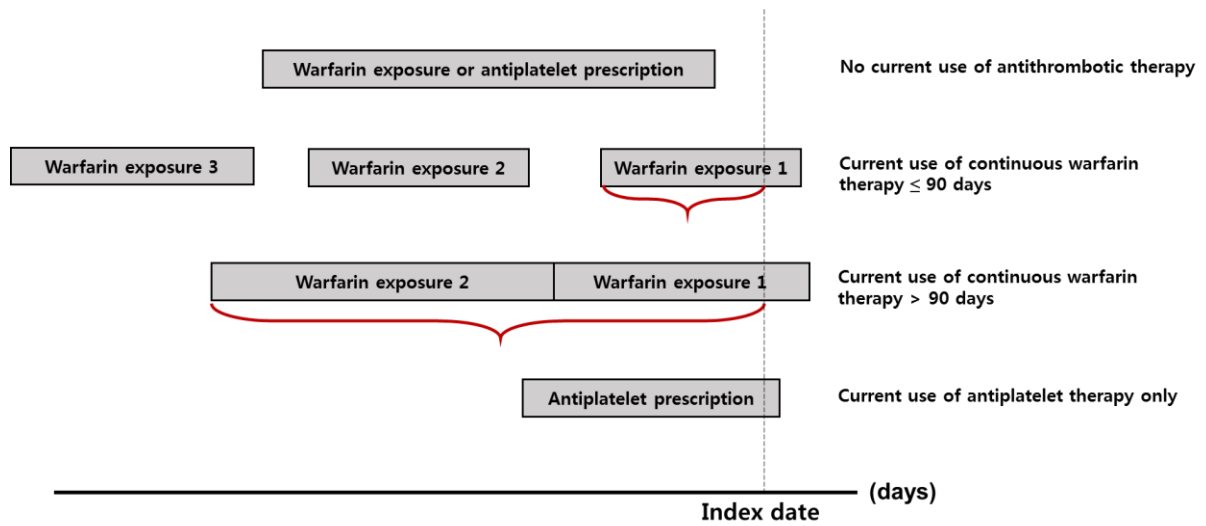

S2 Fig. Current use of continuous antithrombotic therapy

Supplement: S2 Fig — (PDF) [file pone.0179687.s002.pdf]
