# Supplementary material for: Risk of ischemic stroke after atrial fibrillation diagnosis: A national sample cohort
Source: PLoS One. 2017 Jun 21;12(6):e0179687. doi: 10.1371/journal.pone.0179687 (PMC5479557; doi:10.1371/journal.pone.0179687)
Supplement: S3 Fig — (A) CHA2DS2-VASc score and (B) age subgroups. Warfarin treatment was defined as continuous when the warfarin exposure periods (prescription coverage plus 45 days) overlapped each other. (PDF) [file pone.0179687.s003.pdf]

A B

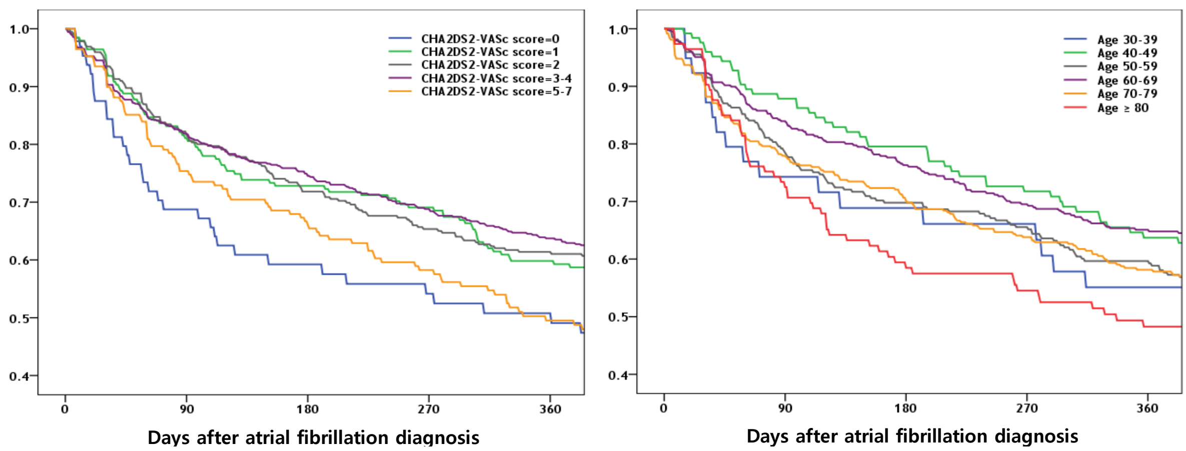

S3 Fig. Proportion of the patients receiving warfarin after atrial fibrillation diagnosis whose warfarin therapy continued during follow-up. (A) CHA_2DS_2-VASc score and (B) age subgroups. Warfarin treatment was defined as continuous when the warfarin exposure periods (prescription coverage plus 45 days) overlapped each other.
