# Supplementary material for: Risk of ischemic stroke after atrial fibrillation diagnosis: A national sample cohort
Source: PLoS One. 2017 Jun 21;12(6):e0179687. doi: 10.1371/journal.pone.0179687 (PMC5479557; doi:10.1371/journal.pone.0179687)
Supplement: S1 Table — The data are reported as n (%). (PDF) [file pone.0179687.s005.pdf]

S1 Table. Frequency of patients in each age subgroup with CHA_2DS_2-VASc scores of 0, 1, 2, 3–4, and 5–7 at atrial fibrillation diagnosis

	**Age, years**						**Total**
	**30–39 (*n*=691)**	**40–49 (*n*=1284)**	**50–59 (*n*=2150)**	**60–69 (*n*=2575)**	**70–79 (*n*=2613)**	**≥80 (*n*=1341)**	**(*n*=10654)**
**CHA_2DS_2-VASc score**							
0	199 (28.8)	286 (22.3)	269 (12.5)	103 (4.0)	0 (0.0)	0 (0.0)	857 (8.0)
1	343 (49.6)	513 (40.0)	738 (34.3)	447 (17.4)	109 (4.2)	0 (0.0)	2150 (20.2)
2	110 (15.9)	307 (23.9)	727 (33.8)	745 (28.9)	388 (14.8)	49 (3.7)	2326 (21.8)
3–4	39 (5.6)	177 (13.8)	410 (19.1)	1157 (44.9)	1455 (55.7)	630 (47.0)	3868 (36.3)
5–7	0 (0.0)	1 (0.1)	6 (0.3)	123 (4.8)	661 (25.3)	662 (49.4)	1453 (13.6)

The data are reported as *n* (%).
